# CO_2_ utilization as a soft oxidant for the synthesis of styrene from ethylbenzene over Co_3_O_4_ supported on magnesium aluminate spinel: role of spinel activation temperature

**DOI:** 10.1038/s41598-020-79188-z

**Published:** 2020-12-17

**Authors:** Venkata Rao Madduluri, Ravi Kumar Marella, Marlia M. Hanafiah, Sivarama Krishna Lakkaboyana, G. Suresh babu

**Affiliations:** 1 Catalysis and Fine Chemicals Laboratory, Indian Institute of Chemical Technology, Hyderabad, 500007 Telangana India; 2 Department of Chemistry (H & S), PACE Institute of Technology and Sciences, Ongole, 523001 Andhra Pradesh India; 3 Department of Earth Sciences and Environment, Faculty of Science and Technology, Universiti Kebangsaan Malaysia (UKM), 43600 Bangi, Selangor Malaysia; 4 Centre for Tropical Climate Change System, Institute of Climate Change, Universiti Kebangsaan Malaysia (UKM), 43600 Bangi, Selangor Malaysia; 5 School of Ocean Engineering, Universiti Malaysia Terengganu, 21030 Kuala Nerus, Terengganu Darul Iman Malaysia; 6 Academy of Scientific and Innovative Research (AcSIR), Sector 19, Kamala Nehru Nagar, Ghaziabad, 20100 Uttar Pradesh India

**Keywords:** Chemistry, Catalyst synthesis, Heterogeneous catalysis

## Abstract

Magnesium aluminate spinel (MgAl_2_O_4_) supported Co_3_O_4_ catalysts are synthesized and tested for the oxidative dehydrogenation (ODH) of ethylbenzene using CO_2_ as a soft oxidant. The effect of spinel calcination temperature on the catalytic performance has been systematically investigated. With an increase in the activation temperature from 600 to 900 °C, the active presence of a single-phase MgAl_2_O_4_ spinel is observed. A catalyst series consisting of MgAl_2_O_4_ spinel with varying Co loadings (10–20 wt%) were prepared and systematically distinguished by ICP, XRD, BET, TPR, NH_3_-TPD, UV–Vis DRS, FT-IR, XPS, SEM, and TEM. Among the tested cobalt catalysts, 15Co/800MA sample derived by calcination of MgAl_2_O_4_ support at 800 °C exhibits the most excellent catalytic performance with the maximum ethylbenzene conversion (≥ 82%). Also, high yields of styrene (≥ 81%) could be consistently achieved on the same active catalyst. Further, the catalyst exhibited almost stable activity during 20 h time-on-stream with a slow decrease in the ethylbenzene conversion from 82 to 59%. However, the selectivity of styrene (98%) stayed almost constant during the reaction. Activation of the MgAl_2_O_4_ spinel at 800 °C facilitates a dramatic chemical homogeneity for the alignment of Co_3_O_4_ nanoparticles on the surface of the active catalyst. Moreover, the isolated Co_3_O_4_ clusters have a strong chemical/electronic interaction with the Mg^2+^ and Al^3+^ ions on the support perform a crucial role to achieve the maximum catalytic activity.

## Introduction

Styrene (ST) monomer is one of the most essential chemical compounds and huge industrial specifications on large scale for the synthesis of several useful commodities. Styrene has been largely synthesized by the oxidative dehydrogenation (ODH) of ethylbenzene (EB) using iron oxide (Fe_2_O_3_) with an excess of steam in the precise range of temperature from 600 to 650 °C^1^. However, there are several problems associated with the ODH of EB like high energy utilization, low yield of ST together with rapid catalyst deactivation, and thermodynamic constraints owing to the endothermic nature. To overcome the above limitations, few efficient catalyst systems are developed by using different oxidizing agents such as O_2_, N_2_O, Ar, and CO_2,_ respectively^2^. Among them, CO_2_ has received more attention due to it stays in gas form during ODH and does not need latent heat for vaporization. In addition, carbon dioxide reduces the reactants partial pressure more effectively in comparison to conventional steam and other oxidants. In other words, CO_2_ utilization helps in the coupling of reverse water–gas shift reaction (RWGSR) with normal dehydrogenation of EB using Fe_2_O_3_/Al_2_O_3_ catalyst^3^. With carbon dioxide as soft oxidant, ODH of EB was studied over the influence of cerium and its precursor on potassium promoted iron catalyst^4^, mesoporous silicalite-1 supported TiO_2_-ZrO_2_^5^, and V_2_O_5_-CeO_2_/TiO_2_-ZrO_2_ catalyst^6^. Chang et al*.* claimed the CO_2_ as active oxidant over supported vanadium-antimony oxide which afforded maximum and steady ST yields compared to steam-assisted process and N_2_ flow conditions^7^. Meso structured nickel-based CeO_2_^8^, and Al_2_O_3_ supported catalysts have been tested for the ODH of EB with good ST yields^9^. Significant EB conversion achieved in the presence of carbon dioxide over CeO_2_–ZrO_2_/SBA-15^10^, MnO_2_–ZrO_2_ and K_2_O/MnO_2_–TiO_2_ catalysts^11^. Significant yield of ST production has been accomplished in the ODH of EB over high-surface-area CeO_2_ catalyst^12^. Park et al*.* published a comprehensive review on the ODH activity of EB over different promoters incorporated on solid oxide catalysts at various reaction conditions^13^. Of late, cobalt nanoparticles receive potential applications in the extensive fields of biomedicine, biotechnology, material engineering, and environmental science^14^. In this scenario, several cobalt-based catalysts are also applied for ODH of EB such as bimetallic Co–Mo catalysts where cobalt-active species have a huge impact on getting high ST production in the presence of CO_2_^15^. Likewise, Moronta et al. reported the use of Co–Mo mixed oxides for the dehydrogenation of EB with a good conversion^16^. Based on the above discussions, reduced catalysts are more efficient for the ODH of EB than supported metal oxides. Our group earlier reported the application of Co_3_O_4_ supported on mixed oxide materials for the oxidative-dehydrogenation of EB under mild reaction conditions^17^. Moreover, cobalt impregnated La/MgO^18^, Co, Ni/carbon nanotube^19^, and Co and Ni ferrites distributed on porous silica catalysts have been utilized in ST synthesis^20^. CoFe_2_O_4_–MCM-41 catalyst is also active for the ODH of EB using CO_2_ as a soft oxidizing agent^21^. In addition to the CO_2_, N_2_ also can play a significant role as co-oxidant when Co-Mo bimetallic nitrides are employed in the ODH of EB^22^.

During the decades, there is a growing concern in the application of magnesium aluminate (MA) spinel as active catalysts or catalyst support in the field of green catalysis, processing of petroleum, and fine-chemicals production. Moreover, the tunable acidic-basic sites, superior chemical strength, and thermal steadiness are crucial for numerous industrial applications^23^. In addition, MgAl_2_O_4_ spinel is also used as an efficient support material for the catalytic steam reforming and dehydrogenation reactions^24,25^. For industrial applications, particularly as catalyst support or catalyst, a high surface area, optimum crystallite size, and a greater number of active sites are significantly preferred^26^. Ji et al*.* developed iron oxide incorporated MA spinel catalysts for ODH of EB, which is beneficial than bulk iron oxide/MgO and iron oxide/γ-Al_2_O_3_ catalysts^27^. Pratap et al*.* reported MgAl_2_O_4_ supported CeO_2_ catalysts with high specific surface area and improved redox properties by co-precipitation method^28^. However, the previously mentioned catalytic systems are not attractive to some extent for desired catalytic activity. So far, ODH of EB over different calcination temperatures of MA spinel supported cobalt oxide catalysts has been unemployed.

In this perspective, for the first time, we are reporting the profound effect of calcination temperature of MgAl_2_O_4_ spinel supported on Co_3_O_4_ catalysts for the ODH of EB at atmospheric pressure using CO_2_ as a soft oxidant. In notable addition, Co_3_O_4_ is used without any pre-reduction, as a result majorly avoids the typical usage of H_2_ which makes it a cost-effective process for industrial application. The growing demand for hydrogen energy makes researchers develop an effective and advanced catalytic process to produce chemical feedstocks^29^. Further, the synthesized samples represent an efficient alternative catalytic system for the ODH of EB than the reported metal oxides including cobalt-based catalysts^15,16^. In the present study, the catalytic performance of Co_3_O_4_/MgAl_2_O_4_ catalysts has precisely correlated with the surface structural characteristics using various analytical and spectroscopic tools as follows.

## Experimental

### Catalyst preparation

Magnesium aluminate spinel (MgAl_2_O_4_) with Mg/Al molar ratio of 1:2 was prepared by the co-precipitation method at a p^H^ of 8.9–9.0, using 5% NH_4_OH solution as a precipitating agent. The precipitate obtained was filtered, gently washed with high purity millipore water followed by oven drying at 100 °C for 12 h. The obtained solid was calcined at different temperatures like 600, 700, 800, and 900 °C for 5 h labeled as 600MA, 700MA, 800MA, and 900MA respectively. A series of cobalt catalysts are prepared by the impregnation method using MgAl_2_O_4_ as support and cobalt nitrate as a metal precursor. The catalysts are calcined at 600 °C for 5 h with a heat ramp of 10 °C/min, designated as x Co_3_O_4_/yMA where ‘x’ indicates weight percentage of cobalt content (x = 10, 15, and 20), whereas ‘y’ represents the calcination temperature of MgAl_2_O_4_ spinel. For the comparison studies, 15 wt% Co_3_O_4_/γ-Al_2_O_3_ (CA) and 15 wt% Co_3_O_4_/MgO (CM) catalysts are synthesized by the wet-impregnation method under similar conditions.

### Characterization of catalyst samples

The XRD experiments for all catalyst samples are carried out in an ULTIMA-IV X-Ray Diffractometer (M/s. Rigaku Instruments, Japan) with Cu Kα monochromatic radiation operating at 40 kV voltage and a current of 30 mA. The crystalline phase of catalysts is determined by continuous scan mode with the 2θ range of 10°–80° at a sampling pitch of 0.07 and 4° min^−1^ scan rate. Reduction patterns of calcined catalysts are investigated by TPR on a self-made reactor system^19^. Typically, 50 mg of the catalyst is loaded in a small quartz reactor and placed in the micro furnace. The reactor is heated linearly up to 800 °C at a ramping rate of 10 °C min^−1^ while flowing 5%H_2_/Ar gas (30 mL/min). After reaching the maximum temperature, the specimen is maintained under isothermal conditions for another 60 min. The amount of H_2_ consumption is monitored by online TCD in-built GC (M/s. CIC Instruments, India)^19^. The amount of cobalt content present in the catalysts was accurately estimated using iCAP 6500 duo ICP-OES Analyzer (M/s. Thermo Fisher Scientific, USA). Before the ICP analysis, the specific cobalt catalyst was dissolved in a stock solution of aquaregia.

FTIR spectra of the samples were recorded on a spectrum-GX spectrometer in the scan range of 4000 to 400 cm^−1^. UV–Vis diffused reflectance spectra (DRS) of samples are investigated on a UV Winlab spectrometer in the UV–Vis region of 200–800 nm. BET principle is employed to investigate the specific surface area of all the catalysts. Before the N_2_ sorption analysis, all samples are degassed under vacuum at 350 °C for 4 h to eliminate physically absorbed gases and moisture. The surface morphology of samples is explored by high-resolution TEM (TECHNAI G2, Japan), operated at an acceleration voltage of 200 kV. A carbon-coated grid is used to disperse the powder which has been mixed with ethanol under sonication. The elementary composition is identified by EDX analysis from the emitted X-rays of the specimen during TEM analysis.

The coke content present in the used catalysts is estimated by TG analysis on TA instruments Q-500 using alumina sample holder heated from 30 to 800 °C at a ramping of 5 °C min^−1^ under O_2_ flow. The NH_3_-TPD studies are performed using ASAP 2020 (M/s. Micrometrics, USA). Typically, 5%NH_3_ balanced He gas is adsorbed at 100 °C for 30 min, then purged with ultra-pure He (20 mL/min) to eliminate the physisorbed water and other gases. The TPD patterns were recorded by desorption of NH_3_ from 100 to 800 °C at a heat ramp of 5 °C min^−1^^30^. The XPS analysis is carried out on a Thermo Scientific K-Alpha Spectrometer operated with Al Kα as X-ray radiation (E_photon_ = 1486.7 eV) at room temperature. The B.E. values of all the elements are accurately calculated by adopting C 1s as a standard reference peak.

## Results and discussion

### Powder X-ray diffraction

**Figure 1:**
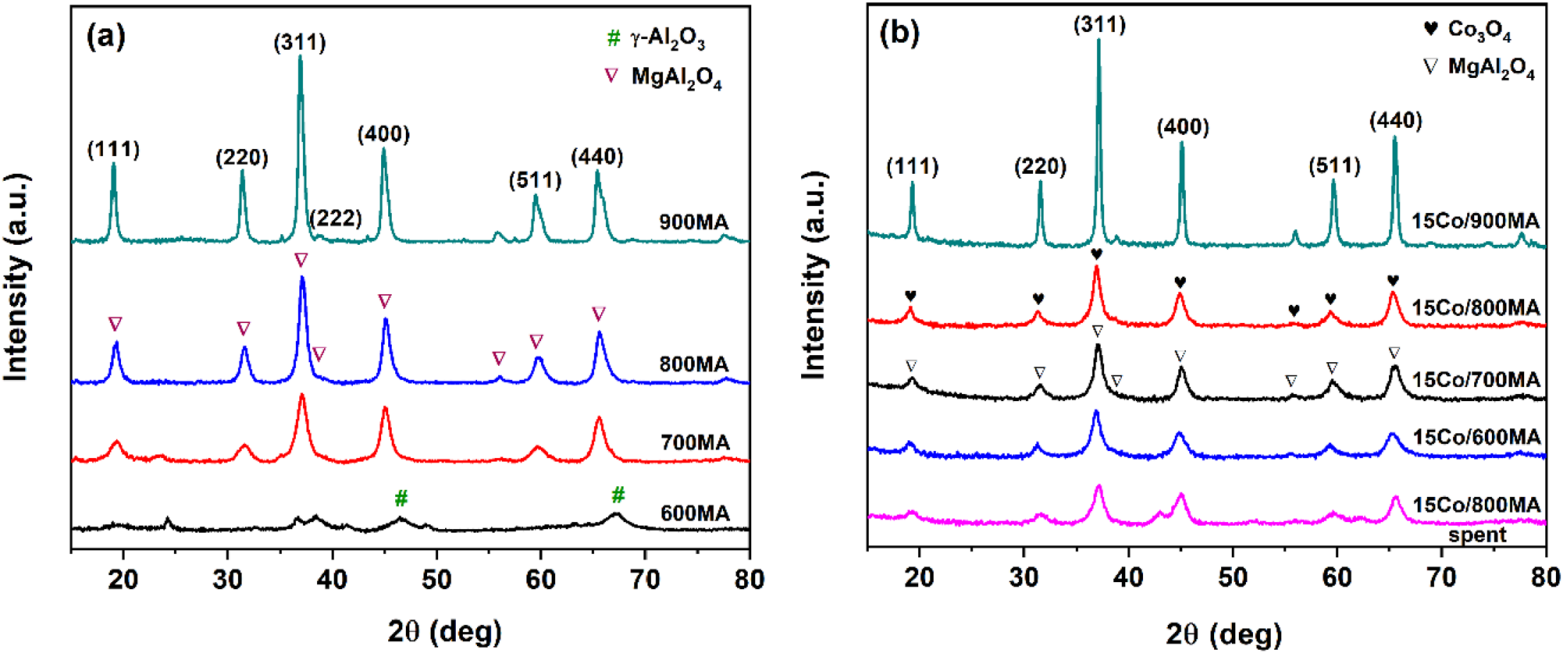
XRD patterns of (**a**) MgAl_2_O_4_ spinel at various activation temperatures and (**b**) cobalt supported catalysts.

The crystallographic structure and active phase of cobalt catalysts with different calcination temperatures of MgAl_2_O_4_ are typically displayed in Fig. 1. The direct XRD reflections corresponding to the lattice planes at (111), (220), (311), (222), (400), (422), (511), and (440) confirm the active presence of single-phase MgAl_2_O_4_ spinel (JCPDS No # 04-008-1061)^31^. As shown in Fig. 1a, the peak intensity of the MgAl_2_O_4_ spinel increases linearly with the gradual rise in calcination temperature, eventually, noticeable growth in crystallite size is typically observed. The 600MA sample exhibited very low-intensity broad peaks due to the partial formation of the MgAl_2_O_4_ spinel phase along with γ-Al_2_O_3_ active phase. The MgO formed by the decomposition of Mg(OH)_2_, reacts instantly with Al(OH)_3_ to form MgAl_2_O_4_ spinel since the Al(OH)_3_ phase is stable up to 600 °C^32^. Conversely, the 700MA sample represents significant growth in the signal intensity, and the unique formation of the MgAl_2_O_4_ spinel phase is observed^33^. It should be carefully noted that when the activation temperature is 800 °C (800MA), a highly pure MgAl_2_O_4_ spinel phase was typically formed. Consequently, a high calcination temperature of 900 °C promoted drastic acceleration in the crystallite size due to severe sintering of MgAl_2_O_4_ spinel particles and typically exhibited an extremely sharp reflection with strong intensity. In summary, calcination in the temperature between 700 to 900 °C is typically preferred for the proper formation of single-phase MgAl_2_O_4_ spinels.

The XRD patterns of MgAl_2_O_4_ supported Co_3_O_4_ catalysts are as shown in Fig. 1b. All the diffractions can be assigned to Co_3_O_4_ (JCPDS No # 00-042-1467) with lattice planes of (111), (220), (311), (222), (400), (422), (511), and (440) respectively^34^. No other reflections are observed, indicating the presence of an active phase with high purity. However, the diffraction lines of Co_3_O_4_ and MgAl_2_O_4_ are not well-resolved which can be attributed to the similar 2θ values of the corresponding active phases^33^. Besides, 15Co/600MA, 15Co/700MA, and 15Co/800MA catalysts display XRD lines with similar intensity while the 15Co/900MA catalyst maintains extraordinary intensity. The spent 15Co/800MA catalyst displayed intact XRD peaks as observed for fresh catalysts even after several hours of activity studies (Fig. 1b). Also, the intensity of direct XRD reflections of 15Co/600MA is higher than the bare 600MA spinel due to the formation of bigger crystallites of Co_3_O_4_ after doping. Similarly, the micropores present in the 900MA spinel are covered by the large Co_3_O_4_ crystals in the 15Co/900MA catalyst, consequently, a drastic change in the crystallite size observed as illustrated in Table 1. However, the crystallite size of the 15Co/700MA catalyst stays unchanged after the doping with Co_3_O_4_. Remarkably, a huge contrast typically emerged between the crystallite size of 800MA spinel and 15Co/800MA catalyst. This can be typically attributed to the uniformly distributed Co_3_O_4_ rather than occupied micropores of the spinel support. A similar trend was also observed in 10Co/800MA and 20Co/800MA catalysts as shown in Fig. S1 (supplementary information).

**Table 1:** Physicochemical characteristics of MgAl_2_O_4_ spinel synthesized at different activation temperatures and its corresponding cobalt supported catalysts.

S. no.	Catalyst	Co loading (wt%)^a^	Crystallite size (nm)^b^	S_BET_ (m^2^/g)^c^	V_p_ (cc/g)^d^	D_p_ (nm)^e^
1	600MA	–	–	152	0.352	8.53
2	700MA	–	20.0	114	0.269	8.24
3	800MA	–	31.0	86	0.135	6.63
4	900MA	–	49.6	52	0.087	6.24
5	15Co/600MA	14.8	16.6	146	0.413	26.2
6	15Co/700MA	14.7	17.5	121	0.362	25.4
7	15Co/800MA	14.8	18.2	144	0.556	32.3
8	15Co/900MA	14.2	62.0	63	0.227	27.5
9	10Co/800MA	9.1	16.8	126	0.479	35.7
10	20Co/800MA	18.2	21.9	136	0.520	31.2
11	15Co/800MA (spent)	14.5	17.0	148	0.523	31.8

^a^Cobalt loading determined from ICP-OES.

^b^Calculated using Scherrer equation by substituting the FWHM value from the lattice plane (311).

^c^Specific surface area of the calcined sample is derived from BET equation.

^d^Total pore volume.

^e^Pore diameter (calculated by BJH method from desorption branch).

### BET surface area and pore-size analysis

The specific BET surface area and pore-size values of as-synthesized MgAl_2_O_4_ spinels at variable calcination temperatures and corresponding Co_3_O_4_ supported catalysts are typically presented in Table 1. It is revealed that with an increase in the calcination temperature of the MA spinel from 600 to 900 °C, there is a noticeable decrease in the surface area from 152 to 52 m^2^/g. This can be ascribed to the generation of bigger crystallites of MgAl_2_O_4_ spinel at more elevated temperatures follwing the XRD results. The high surface area of the 600MA sample is majorly due to the possible formation of γ-Al_2_O_3_ phase along with MgAl_2_O_4_ spinel below the activation temperature of 700 °C^33^. In addition, the pore volume and pore diameter of the samples also decreased with rising the calcination temperature owing to the gradual increase in the crystallite size of MA spinel.

However, the Co_3_O_4_ doped MgAl_2_O_4_ spinel catalysts have typically exhibited fascinating results as mentioned in Table 1. The addition of nominal cobalt content to the MA support forms a uniform layer of Co_3_O_4_ during the thermal treatment. This phenomenon results increase in the specific surface area and pore-size of the Co_3_O_4_/MgAl_2_O_4_ samples when compared with the bare support. From Table 1, the measured surface area of 15Co/600MA and 15Co/700MA catalysts are 146 and 121 m^2^/g respectively. The smooth decrease in the surface area probably due to the partial filling of Co_3_O_4_ species in the micropores of the MgAl_2_O_4_ spinel. Remarkably, the high surface area and pore-size of 15Co/800MA catalyst can be explained based on the fine dispersion of Co_3_O_4_ particles on the 800MA support. Likewise, 10Co/800MA and 20Co/800MA catalysts promptly follow a similar trend and typically exhibited a more specific surface area as stated in Table 1.

In contrast, the low surface area of the 900MA spinel could not provide enough external surface space for the sheer distribution of Co_3_O_4_ clusters. Therefore, the 15Co/900MA catalyst has typically shown an extremely low specific surface area in comparison with the other samples. As per the above discussion, 15Co/800MA represents the best catalyst for the titled reaction with a high specific surface area and large pore size.

### H_2_-TPR

**Figure 2:**
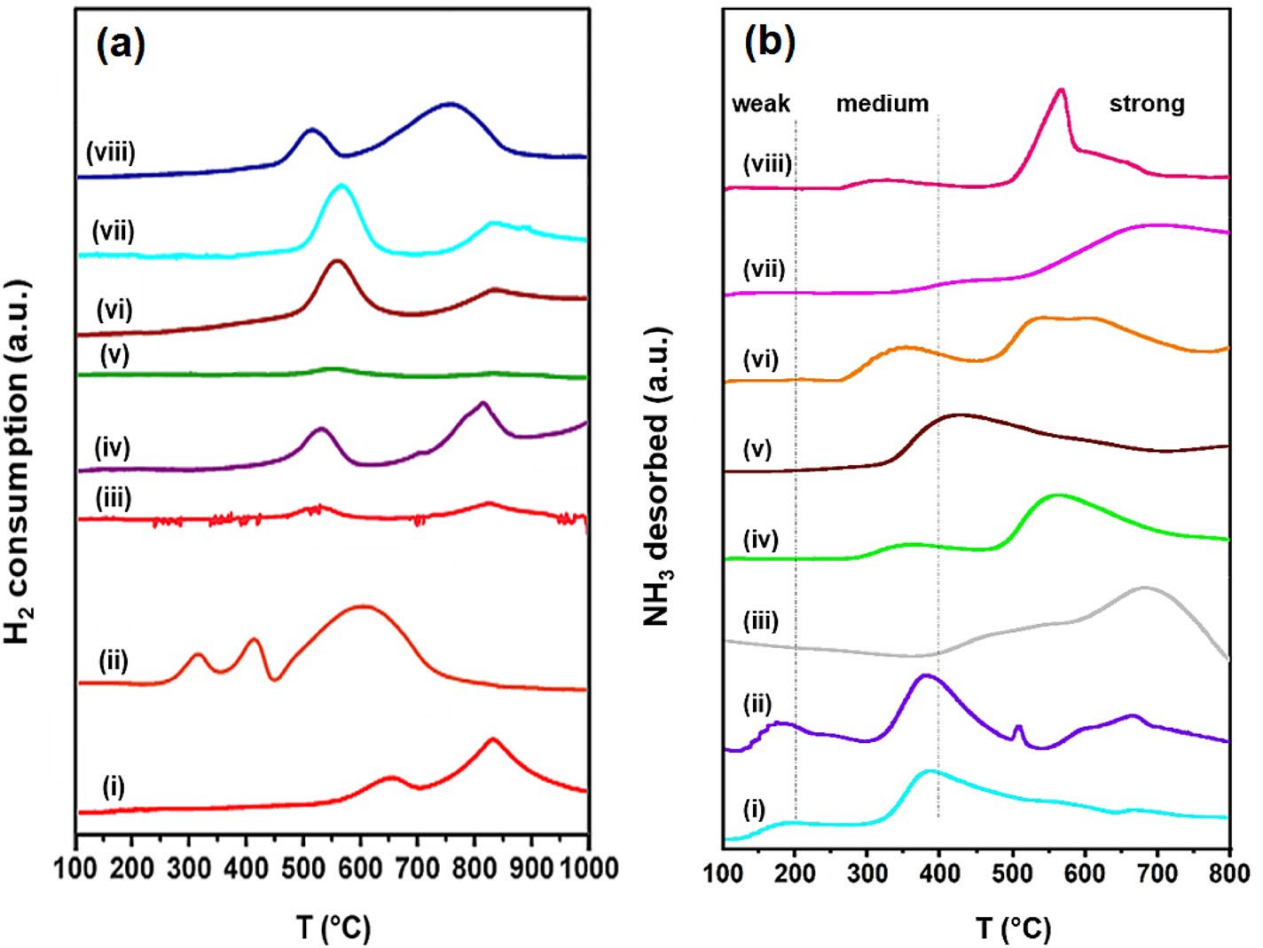
(**a**) H_2_-TPR patterns of (i) CA, (ii) CM, (iii) 15Co/600MA, (iv) 15Co/700MA, (v) 10Co/800MA, (vi) 15Co/800MA, (vii) 20Co/800MA, and (viii) 15Co/900MA catalysts. (**b**) NH_3_-TPD profiles of (i) 800MA, (ii) CM, (iii) CA, (iv) 15Co/700MA, (v) 10Co/800MA, (vi) 15Co/800MA, (vii) 20Co/800MA, and (viii) 15Co/900MA catalysts.

The reduction profiles of Co/MA spinel catalysts including 15Co/γ-Al_2_O_3_ (CA), and 15Co/MgO (CM) are typically shown in Fig. 2a. It has been extensively investigated that the reduction pattern of Co_3_O_4_ could be influenced by several factors like preparation method, metal precursor, nature of the support, and activation treatment. The TPR profiles of CA, CM, Co/MA (i.e., 15Co/700MA, and 15Co/900MA) catalysts exhibit the reduction peak with high intensity at elevated temperatures compared to those present in low-temperature reduction signals. This allows a scope to think that the reduction process of Co_3_O_4_ phase in 15Co/γ-Al_2_O_3_, 15Co/MgO, 15Co/700MA, and 15Co/900MA catalysts proceed in two steps as follows:

1 $$ {\text{Co}}_{{3}} {\text{O}}_{{4}} + {\text{ H}}_{{2}} \to {\text{3CoO }} + {\text{ H}}_{{2}} {\text{O}} $$

2 $$ {\text{3CoO }} + {\text{ 3H}}_{{2}} \to {\text{3Co }} + {\text{ 3H}}_{{2}} {\text{O}} $$

Thereby, H_2_ consumption could be higher for 15Co/900MA catalyst because of larger crystallite size than 10Co/800MA, 15Co/800MA and 20Co/800MA catalysts. The H_2_-TPR of 15Co/700MA catalyst displayed two reduction signals one at a lower temperature (500–600 °C) with less intensity and another peak at a high temperature (600–900 °C) with more intensity. The low-temperature signal can be ascribed to the reduction of uniformly distributed Co_3_O_4_ particles. Whereas, the high-temperature peak is attributed to the complex reduction pattern of strongly interacted Co_3_O_4_ species. Besides, the 15Co/γ-Al_2_O_3_ catalyst exhibited two major reduction peaks at high temperature due to the strong interaction of Co_3_O_4_ species with γ-Al_2_O_3_ support^35^. It is reported that transition metal (M^2+^) ions preferentially occupies tetrahedral vacancies of γ-Al_2_O_3_, so it is difficult to get metallic Co, compared to those occupied in octahedral vacancies of γ-Al_2_O_3_^36^. In the case of Co/MgO catalyst three different reduction signals are observed with different peak intensities as described in Fig. 2a. Amongst, the first reduced signal centered at 300 °C is ascribed to the direct transition of Co_3_O_4_ into metallic Co species, while second reduced signal about T_max_ 400 °C is illustrated the transformation of strongly support interacted Co_3_O_4_ species into CoO and metallic Co. Then, reduction pattern at high temperature (600 °C) may probably represent the formation of some solid solution species such as MgCo_2_O_4_ and CoO-MgO species in Co_3_O_4_/MgO catalyst^37^.

However, reduction patterns of 10Co/800MA, 15Co/800MA, and 20Co/800MA catalyst are quite different from Co/MgO, Co/γ-Al_2_O_3_, 15Co/700MA, and 15Co/900MA catalyst. As a result, 10Co/800MA, 15Co/800MA, and 20Co/800MA catalysts displayed facile reduction patterns owing to synergistic interface between Co_3_O_4_ and MgAl_2_O_4_ support. Therefore, 15Co/800MA catalyst is exhibited low-intense reduced signals compared to those present in Co/MgO, Co/γ-Al_2_O_3_, 15Co/700MA, 20Co/800MA, and 15Co/900MA catalysts. Furthermore, the surface textural properties of 15Co/800MA catalyst is better in line with smaller crystallite size, and high surface area facilitates the easy reduction of Co_3_O_4_ species as evidenced from H_2_-TPR patterns (Fig. 2a). In the present investigation, reduction patterns of 10Co/800MA and 20Co/800MA catalysts are almost identical with the 15Co/800MA catalyst. In notable addition, highly intense TPR signals are observed for 20Co/800MA catalyst due to the reduction of bulk Co_3_O_4_ species. While 10Co/800MA catalyst displayed very low intensity TPR signals owing to the low cobalt oxide content. Similarly, the 15Co/600MA catalyst contain two reduction peaks as observed in 10Co/800MA catalyst, which is in agreement with the reported literature^35,38^.

### UV–Vis DRS

The UV–Vis spectra of MgAl_2_O_4_ spinel activated at various temperatures are presented in Fig. S2 (supplementary information), where the K–M functions of the bands are plotted as a function of wavelength. It is evident from the figure that absorption bands of all samples are following a similar trend with small variation in the peak intensity. The absorption maxima below 300 nm can be attributed to the charge transfer (CT) from O^−2^ to Al^3+^ ions due to excitation of electrons from the valence band of O(2p) to the conduction band of Al(3d)^31^.

**Figure 3:**
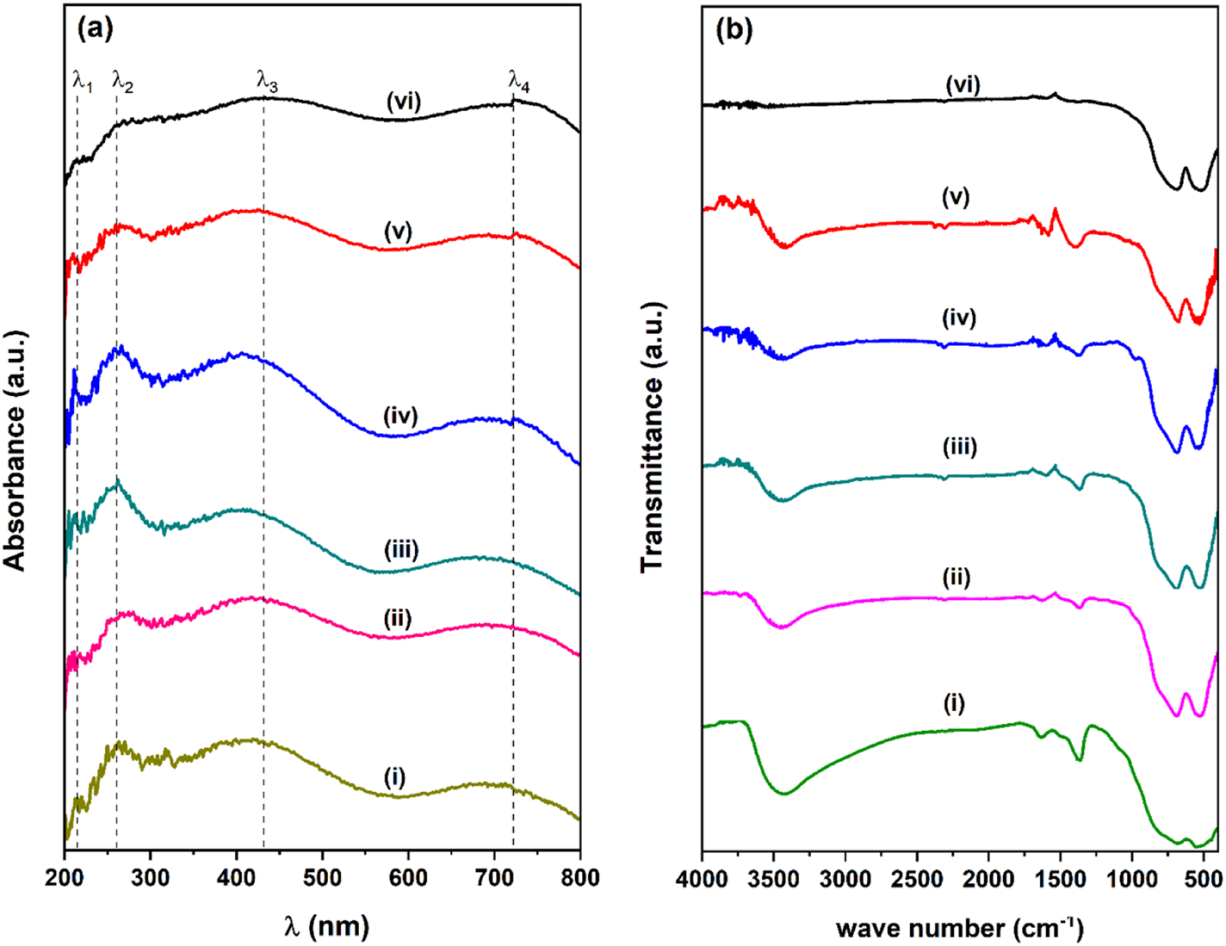
(**a**) UV–Vis spectral analysis of (i) 15Co/600MA (ii) 15Co/700MA (iii) 10Co/800MA (iv) 15Co/800MA (v) 20Co/800MA, and (vi) 15Co/900MA catalysts. (**b**) FTIR spectral analysis of (i) 15Co/600MA (ii) 15Co/700MA (iii) 10Co/800MA (iv) 15Co/800MA (v) 20Co/800MA, and (vi) 15Co/900MA catalysts.

As shown in Fig. 3a, a split in the absorption bands below 300 nm observed for Co_3_O_4_ supported catalysts placed at 214 nm (λ_1_) and 240 nm (λ_2_) corresponding to the CT transition of O^−2^ → Al^3+^ and O^−2^ → Co^3+^ respectively^39,40^. The broad absorption signals centered at 430 (λ_3_) nm and 720 nm (λ_4_) among all the cobalt catalysts indicate the presence of a large amount of Co atoms in the octahedral and tetrahedral symmetry respectively^41^. In general, CoAl_2_O_4_ spinel might be formed during high-temperature due to diffusion of Co^2+^ ions into the alumina, and their distribution possibly varies with the amount of Co loading in the lattice. However, no peaks corresponding to tetrahedral Co^2+^ symmetry (~ 620 nm) confirms the absence of the CoAl_2_O_4_ spinel phase among all the MgAl_2_O_4_ supported cobalt catalysts.

### FT-IR

The FT-IR spectra of MgAl_2_O_4_ spinel synthesized at various calcination temperatures were recorded in the frequency range from 4000 to 400 cm^−1^ and illustrated in Fig. S2 (supplementary information). The visible FTIR bands in a range of 900 to 500 cm^−1^ can be attributed to the metal–oxygen (M–O) stretching vibrations, where “M” denotes either Al or Mg. The signal at 700 cm^−1^ indicates the presence of Al^3+^ ions in the octahedral sites, and another vibration band at 750 cm^−1^ assigned to the occupation of Mg^2+^ ions in the tetrahedral sites^28^. Therefore, can be confirmed the formation of a single-phase MgAl_2_O_4_ spinel with rising the activation temperature from 600 to 900 °C. Further, the signal located at 1630 cm^−1^ is assigned to the deformation band of interlayer water molecules (H–O–H) corresponding to the MgAl_2_O_4_ spinel surface^28^. The broad FTIR band at 3430 cm^−1^ could be plausibly attributed to the O–H stretching vibration of the surface adsorbed water molecules.

The FTIR spectral analysis of Co_3_O_4_ incorporated on MgAl_2_O_4_ spinel catalysts prepared by the impregnation method is typically displayed in Fig. 3b. The Co–O stretching vibration bands typically displayed at an optimal frequency of 570 and 675 cm^−1^ which are ascribed to the octahedral and tetrahedral symmetry of Co^3+^ ions^42^. However, no apparent difference is found between the FTIR spectra of Co/MA catalysts and MA support, because of the overlapping of Co_3_O_4_ signals with MgAl_2_O_4_ spinel.

### NH_3_-TPD

To explore the distribution of the surface acidic sites on MgAl_2_O_4_ spinel (MA) including MA supported cobalt catalysts, NH_3_-TPD was performed as illustrated in Fig. 2b. The resulting basic sites analyzed from TPD patterns of samples are further classified into three categories (i.e., weak, moderate, and strong) assigning to their desorption strength. However, the bare 800MA support contains a lesser number of strong acidic sites, although the uniform distribution of weak and moderate acidic sites is investigated because of balanced Al^3+^ and Mg^2+^ ions. In the CM catalyst, weak and moderate acidic sites are massive in number, whereas strong acidic sites are relatively lesser compared to that of CA catalysts. It is majorly owing to the more significant number of Mg^2+^ species present in CM catalyst; consequently, diminish the surface acidic nature of the catalyst. Moreover, the formation of some extent solid solution species like MgCo_2_O_4_ also decreases the surface acidic nature of CM catalyst. Therefore, CM catalyst afforded a low conversion of EB but promoted high styrene selectivity as illustrated in Table 3.

In the case of CA, 10Co/800MA, 15Co/800MA, and 20Co/800MA catalysts strong acidic site distribution and the total acidity are relatively higher than CM, 15Co/900MA, and 800MA samples as mentioned in Table 2. The 10Co/800MA catalyst displayed a lower fraction of moderate and strong acidic sites, because of less cobalt oxide content. In contrast, 20Co/800MA catalyst with more cobalt oxide containing a more considerable fraction of strong acidic sites accordingly severe decrease of styrene monomer selectivity (Table 3). Whereas, a more significant quantity of weak and moderate acidic sites along with strong acidic sites exist in the CA catalyst is responsible for poor catalytic activity. In a similar approach, less acidity present in the 800MA and CM samples is unfavorable for high EB conversion and ST selectivity (Table 3). The 15Co/900MA catalyst despite containing less acidic sites did not yield more ST owing to the sintering of active cobalt oxide particles at high calcination temperatures. The number of weak and moderate acidic sites generated in 15Co/800MA catalyst is considerably higher in number due to the uniform alignment of Co_3_O_4_ species, but strong acidic sites are noticeably low compared to CA and 20Co/800MA catalyst.

**Table 2:** NH_3_-TPD analysis of MgAl_2_O_4_ (MA), 15Co_3_O_4_/γ-Al_2_O_3_ (CA), 15Co_3_O_4_/MgO (CM), and Co_3_O_4_/MgAl_2_O_4_ (Co/MA) samples.

S. No.	Sample	Weak (100–200 °C)	Moderate (200–400 °C)	Strong (400–800 °C)	Total acidity (mmol. g^−1^)
1	800MA	0.032	0.158	0.352	0.510
2	CM	0.085	0.312	0.492	0.889
3	CA	0.021	0.106	1.026	1.153
4	15Co/700MA	–	0.159	0.472	0.631
5	10Co/800MA	–	0.185	0.491	0.676
6	15Co/800MA	–	0.257	0.655	0.912
7	20Co/800MA	–	0.126	1.194	1.320
8	15Co/900MA	–	0.139	0.381	0.520

**Table 3:** ODH of EB over MgAl_2_O_4_ (MA), Co_3_O_4_/MgAl_2_O_4_ (Co/MA), 15Co_3_O_4_/γ-Al_2_O_3_ (CA), and 15Co_3_O_4_/MgO (CM) samples in CO_2_ atmosphere.

S. No.	Catalyst	X_EB_ (%)^a^	S_ST_ (%)^b^	S_others_ (%)^c^
1	600MA	29	92	8
2	700MA	35	96	4
3	800MA	42	98	2
4	900MA	23	97	3
5	15Co/600MA	64	94	6
6	15Co/700MA	71	95	5
7	10Co/800MA	63	95	5
8	15Co/800MA	82	98	2
9	20Co/800MA	74	87	13
10	15Co/900MA	56	93	7
11	CA	52	91	9
12	CM	46	98	2

^a^X_EB_ = conversion of EB.

^b^S_ST_ = selectivity of ST.

^c^S_others_ = selectivity of other by-products.

*Reaction conditions: Catalyst = 0.7 g, T = 600 °C, CO_2_ flow = 30 mL/min, EB flow = 1.5 mL/h.

Typically, based on NH_3_-TPD patterns neither a low fraction of acidic sites nor strong acidic sites generated are suitable for potential catalytic activity. Therefore, balanced weak and moderate acidic nature along with adequate strong acidic sites possessed 15Co/800MA catalyst represented a significant role in getting maximum EB conversion and styrene selectivity.

### XPS

**Figure 4:**
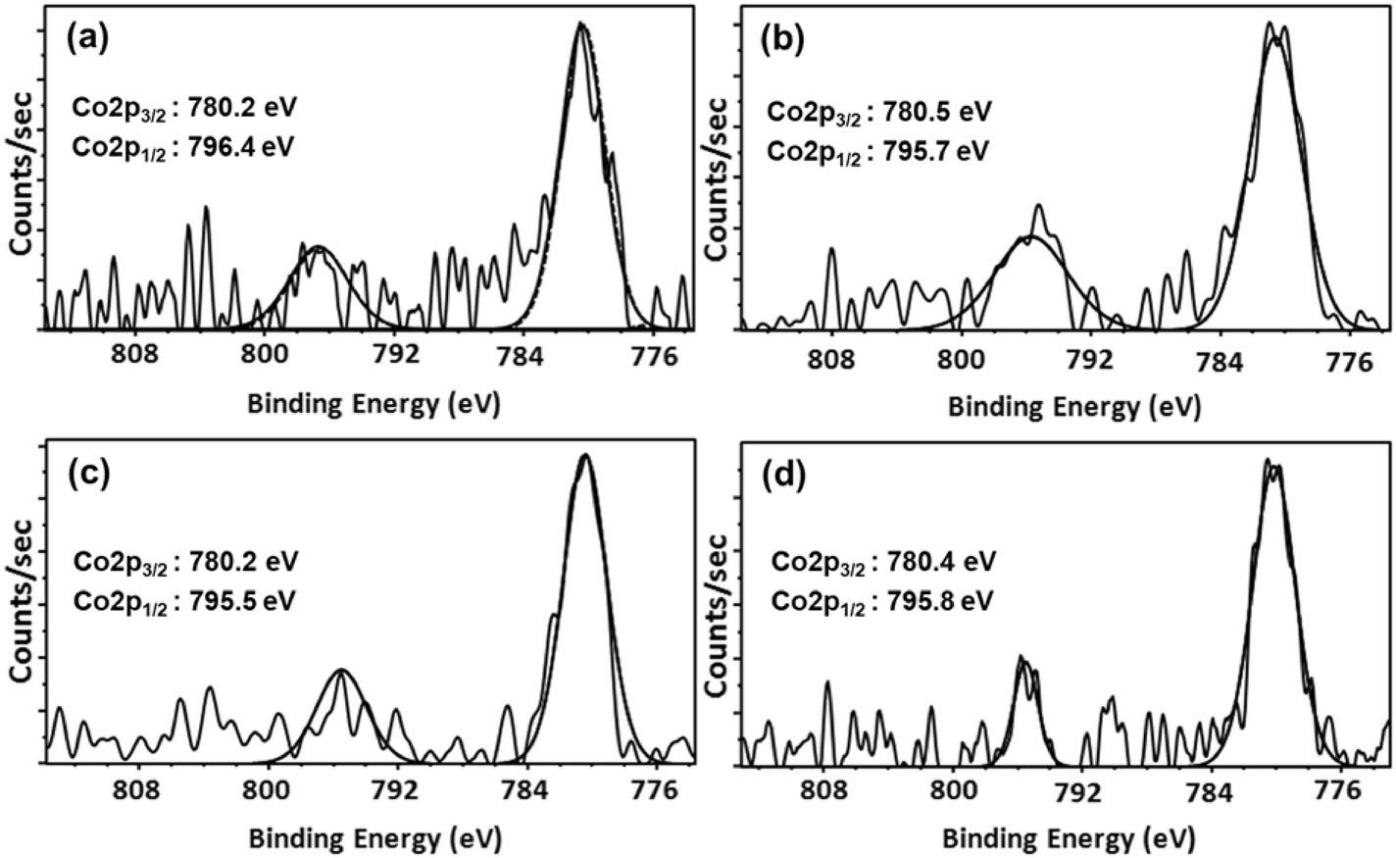
Co2p XPS spectra of (**a**) 15Co/600MA, (**b**) 15Co/700MA, (**c**) 15Co/800MA and (**d**) 15Co/900MA catalysts.

The oxidation state of active cobalt species present in the calcined Co/MA catalysts is detected by the XPS technique and depicted in Fig. 4. The binding energy (B.E.) value of Co2p_3/2_ at ~ 780 eV and Co2p_1/2_ at ~ 795 eV with a spin-orbital splitting of 15.2 eV and the absence of intense satellite peaks demonstrate the presence of Co_3_O_4_ species among all the cobalt catalysts^43–45^. The B.E. value of Al2p at 74.1 eV and Mg1s at 1303.4 eV indicates the presence of Al^+3^ and Mg^+2^ ions in the form of MgAl_2_O_4_ spinel (Fig. S3, supplementary information)^40^. Nevertheless, the 15Co/800MA catalyst exhibited a lower B.E. value than 15Co/700MA, 15Co/900MA, and 20Co/800MA catalysts owing to the synergetic interaction between Co_3_O_4_ and MgAl_2_O_4_ support^41^.

### TEM

**Figure 5:**
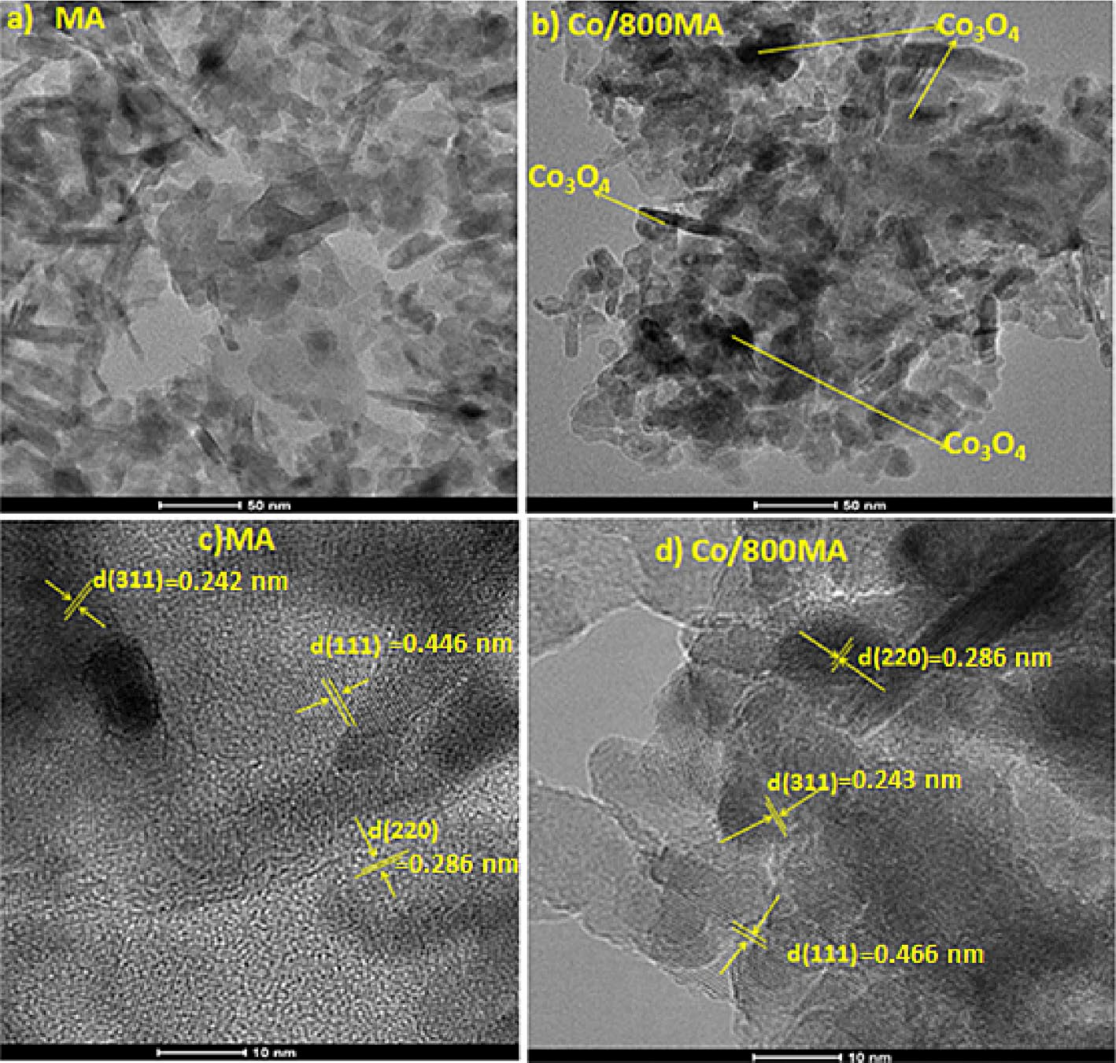
HRTEM images of 800MA spinel (MA) and 15Co/800MA catalysts.

HRTEM analysis of MgAl_2_O_4_ spinel (MA) and 15Co/800MA catalyst are illustrated in Fig. 5. As shown in Fig. 5b, 15Co/800MA catalyst displayed homogeneously distributed nano-sized cobalt oxide particles environment on the high surface area of MA spinel. Similarly, the bare 800MA spinel (Fig. 5c) is prominently displayed a lesser number of aggregated species with related lattice fringes substantially similar to the 15Co/800MA catalyst. As shown in Fig. 5d, the lattice resolved HRTEM image of 15Co/800MA catalyst confirm the uniformly distributed nano-sized Co_3_O_4_ particles through crystal planes of (111), (311) and (220) with d-spacing of 0.466, 0.243 and, 0.286 nm respectively^46^. However, the majority of the cobalt oxide fringes are typically covered over the surface of MA spinel. Further, SAED patterns authenticate the amorphous or poor crystalline nature of 800MA spinel and 15Co/800MA catalyst as outlined in Fig. S4 (supplementary information). Indeed, the SAED pattern of 800MA spinel showed bright circular planes compared to low-intensity circular planes in the SAED image of 15Co/800MA catalysts.

The elementary composition of Co, Mg, and Al present in the fresh and spent 15Co/800MA catalysts are illustrated in the supplementary information (Figs. S5 and S6). It can be observed that no appreciable leaching of active cobalt oxide present in the used catalyst even after several hours of catalytic activity. The EDX elementary mapping of HAADF-STEM images of 15Co/800MA catalyst is as shown in Fig. S7 (supplementary information). It can be distinguished that a more considerable number of isolated and uniformly distributed Co_3_O_4_ species on the surface of the MgAl_2_O_4_ support. All these consistent findings are precisely following the HRTEM morphology results procured as illustrated in Fig. 5.

### Thermogravimetric analysis

TG analysis of spent cobalt catalysts collected after 20 h of EB dehydrogenation activity is graphically displayed in the supplementary information (Fig. S8). As shown in the figure, TGA patterns of 15Co/700MA, 15Co/800MA, and 15Co/900MA catalysts exhibit a characteristic signal in the temperature range of 400–500 °C in all the catalysts. This can be attributed to the decomposition of deposited hydrocarbon moiety^28^. The 15Co/800MA and 15Co/700MA catalysts have a low intense endothermic signal beyond 450 °C with a weight loss of 10% and 11.5% results from the catalytic oxidation of deposited carbon (coke). While 15Co/900MA catalyst has a steep decomposition peak at 470 °C with a significant weight loss of 14.5% owing to excessive decomposition of EB molecules on the cobalt oxide particles with large crystallize size. Also, elemental analysis results are in line with the TGA analysis concerning coke deposition on spent catalysts. As described in TGA patterns, less coke deposition is investigated on the surface of 15Co/800MA spent catalysts because of efficient oxidation of deposited carbon into CO_2_ molecules in the course of ODH of EB. Therefore, one can expect that the 15Co/800MA catalyst would typically exhibit a steady catalytic activity compared to 15Co/700MA and 15Co/900MA catalyst.

### Catalytic activity studies

The CO_2_ assisted ODH of EB over cobalt catalysts takes place in two steps as shown in Eq. (3) and (4). In the first step, EB dehydrogenated into ST with the liberation of H_2_. Afterward, the H_2_ formed will typically react with CO_2_ via reverse water–gas shift (RWGS) reaction. Therefore, the general reaction comprises a combination of EB dehydrogenation and RWGS reaction as displayed in Eq. (5).

3 $$ {\text{EB}} \to {\text{ST }} + {\text{ H}}_{{2}} $$

4 $$ {\text{CO}}_{{2}} + {\text{ H}}_{{2}} \to {\text{CO}} + {\text{H}}_{{2}} {\text{O}} $$

5 $$ {\text{EB }} + {\text{ CO}}_{{2}} \to {\text{ST}} + {\text{CO}} + {\text{H}}_{{2}} {\text{O}} $$

### Influence of temperature on the ODH of EB activity

**Figure 6:**
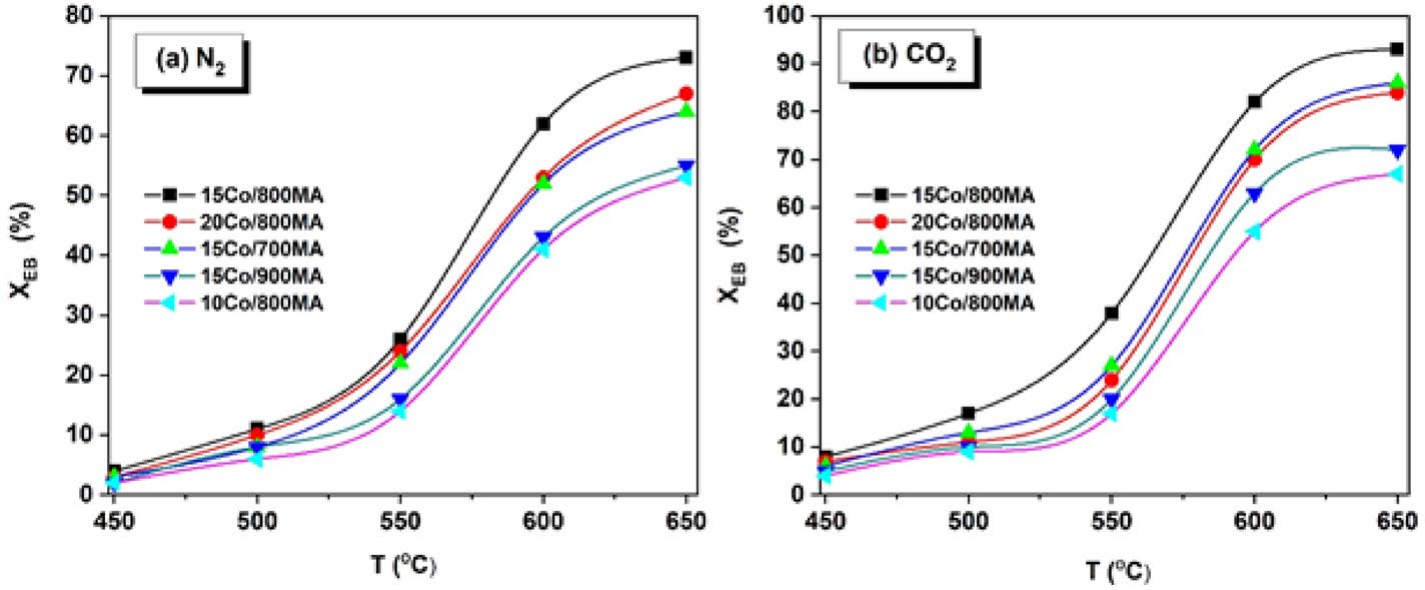
Influence of temperature on EB conversion over Co/MA catalysts under (**a**) N_2_, and (**b**) CO_2_ (conditions: T = 450–600 °C, catalyst = 0.7 g, N_2_ (or) CO_2_ = 30 mL/min, EB = 1.5 mL/h).

The influence of reaction temperature on the EB conversion (X_EB_) was investigated in the presence of N_2_ and CO_2_ as oxidants over Co/MA catalysts as depicted in Fig. 6a,b respectively. Typically, ODH of EB is endothermic thereby mostly depends on the reaction temperature^47^. Therefore, with a rise in the temperature from 450 to 650 °C, X_EB_ increased gradually to a maximum extent. As evident from Fig. 6, it can be observed that the remarkable activity of CO_2_ assisted ODH of EB in comparison with the EB dehydrogenation under N_2_ flow.

Among all the catalysts, 15Co/800MA catalyst performed outstanding activity than other samples (Fig. 6b), where the X_EB_ increased progressively from 8 to 82% with a change in the temperature from 450 to 600 C. Further increase in the reaction temperature to 650 °C, which showed a maximum X_EB_ (93%) but the selectivity of ST decreased to significant levels (not shown in Fig. 6). It can be observed that all the samples afforded very mild activity of EB at low reaction temperatures (450–550 °C), and then considerable improvement in X_EB_ is observed at 600 °C. After recognizing the optimum temperature, further CO_2_ assisted ODH of EB reactions were carried out over all the cobalt catalysts including the MA support and the results are illustrated in Table 3.

MA spinel activated at low calcination temperature (600MA) exhibits a very low X_EB_ (29%) and S_ST_ (92%), which can be ascribed to the partial formation of single-phase MgAl_2_O_4_ spinel. In contrast, improved X_EB_ of 700MA (35%), and 800MA (42%) owing to the complete formation of single-phase MA spinel. However, the X_EB_ of 900MA spinel remained at 23% owing to the sintering of Mg^2+^ and Al^3+^ ions result in the formation of bigger crystallites with low specific surface area as depicted in Table 1.

After the incorporation of the optimal amount Co_3_O_4_ onto the 800MA spinel support, the catalytic activity increased two folds from 42 to 82%. Thus, cobalt oxide nanoparticles played an active role via synergistic interaction with the MgAl_2_O_4_ spinel support. Moreover, homogeneously distributed Co_3_O_4_ species on 800MA spinel with adequate surface acidic-basic sites has shown high EB conversion and ST selectivity. Among all, 15Co/600MA catalyst displayed the lowest X_EB_ and S_ST_ because of the lesser number of Co_3_O_4_ clusters on the MgAl_2_O_4_ spinel support and the presence of γ-Al_2_O_3_ phase as described in XRD (Fig. 1a). Besides, 15Co/700MA and 20Co/800MA catalysts with almost similar physicochemical characteristics (Table 1) afforded some extent equal X_EB_ at all reaction temperatures. The high surface area, thermal stability, and optimum surface acidic nature of the 15Co/800MA sample provide an enhanced chemical homogeneity towards the uniform distribution of Co_3_O_4_ nanoparticles. As a result, less coke deposition is observed on the external surface of the 15Co/800MA spinel catalyst even after several hours of catalytic activity study when compared to 15Co/700MA and 15Co/800MA catalysts, further evidenced from TGA analysis as displayed in Fig. S8 (supplementary information). It can be observed that neither low acidic strength of 10Co/800MA catalyst nor strong acidic sites of 20Co/800MA catalyst are suitable for achieving optimum catalytic activity (Table 3). Similarly, a very low specific surface area, and large crystallite size 15Co/900MA catalyst is also undesirable for the higher activity of CO_2_ assisted ODH of EB.

### Time-on-stream (TOS) study

**Figure 7:**
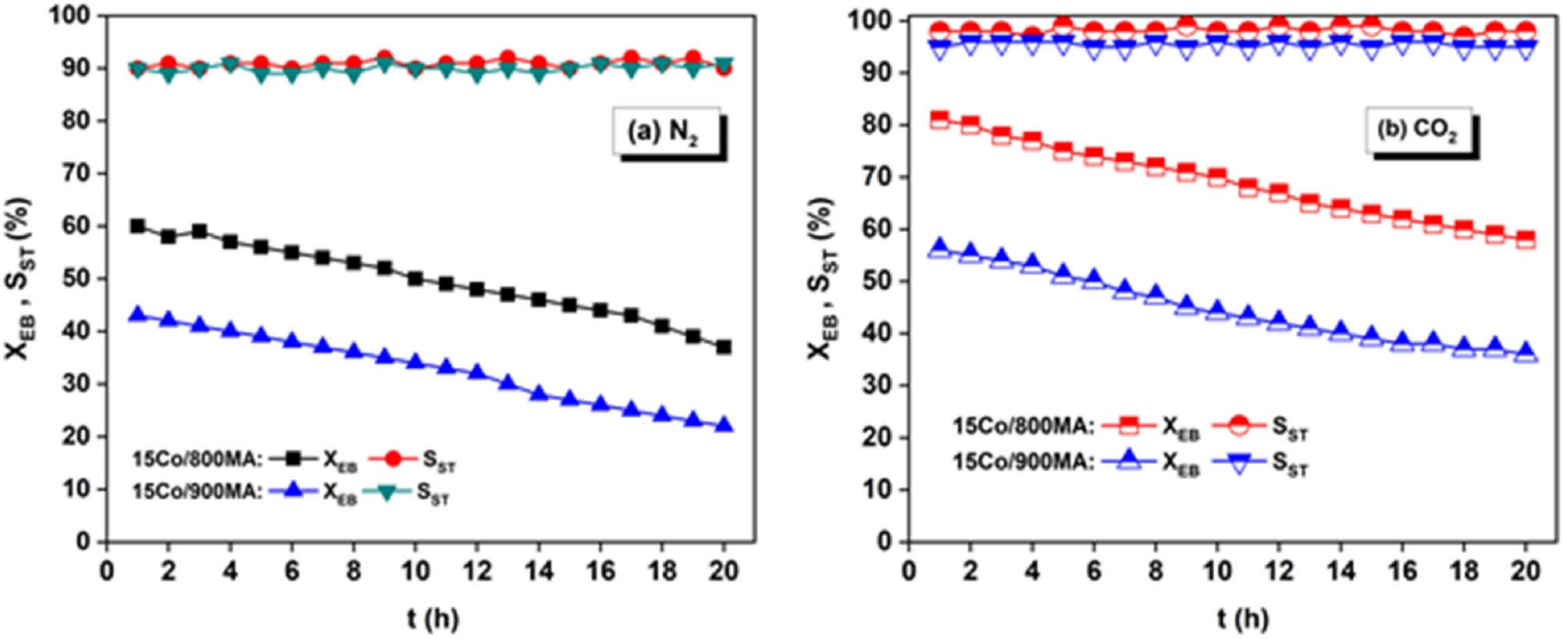
TOS on 15Co/800MA and 15Co/900MA catalysts under (**a**) N_2_ flow, (**b**) CO_2_ flow (**a**) N_2_ flow, (**b**) CO_2_ flow (conditions: T = 600 °C, catalyst = 0.7 g, N_2_ = 30 mL/min, EB = 1.5 mL/h).

To distinguish the long-term stability for the ODH of EB, TOS studies are performed over 15Co/800MA and 15Co/900MA catalysts in the presence of N_2_ and CO_2_ atmosphere as drawn in Fig. 7. It is evident from Fig. 7a, with an increase in the reaction time from 1 to 20 h, the X_EB_ decreases from 60 to 37% over the 15Co/800MA catalyst. Similarly, X_EB_ decreases from 43 to 23% over the 15Co/900MA catalyst with passing time. However, the S_ST_ stayed constant at 90% in both the samples during the reaction. Typically, the generated ST undergoes self-polymerization to yield polystyrene, which is crucial for coke deposition on the active surface of the catalyst^12^. In the presence of N_2_, the polystyrene will transform into graphitic carbon at elevated temperature (> 500 °C), consequently, rapid catalyst deactivation takes place (Fig. 7a).

It can be observed the X_EB_ decreases from 82 to 59% over the 15Co/800MA catalyst the 15Co/900MA catalyst follows a similar trend where the X_EB_ declined from 58 to 37%. However, the S_ST_ of 15Co/800MA (98%) and 15Co/900MA (95%) remains constant throughout the catalytic run. It is supposed to be due to the promotion of the RWGSR phenomenon in the presence of the CO_2_ environment, which played a key role in superior catalytic activity. Besides, a greater number of uniform Co_3_O_4_ oxide particles on the 800MA spinel results in the high specific surface area and optimum crystallite size responsible for the enhancement in the catalytic activity. Nevertheless, the coke formation can be removed effectively by the oxidation of CO_2_ as a soft oxidant. Hence, CO_2_ assisted ODH of EB has a potential role to improve the catalytic activity as evident from Fig. 7b.

In the case of 10Co/800MA catalyst, a mild fraction of Co_3_O_4_ oxide particles leads to the marginal conversion of EB but no major changes are observed in the ST selectivity. In contrast, high Co oxide content possessed by 20Co/800MA catalyst influence the ST selectivity due to the high density of surface acidic sites as evidenced from NH_3_-TPD analysis (Table 2). For convenience, we have not included the TOS study of 10Co/800MA and 20Co/900MA catalysts in the manuscript, however, corresponding X_EB_ and S_ST_ are listed in Table 3. In contrast, 15Co/900MA catalyst accomplished insignificant X_EB_ during the time-on-stream owing to the bigger crystallite size and subsequently displayed low surface area as listed in Table 1.

**Figure 8:**
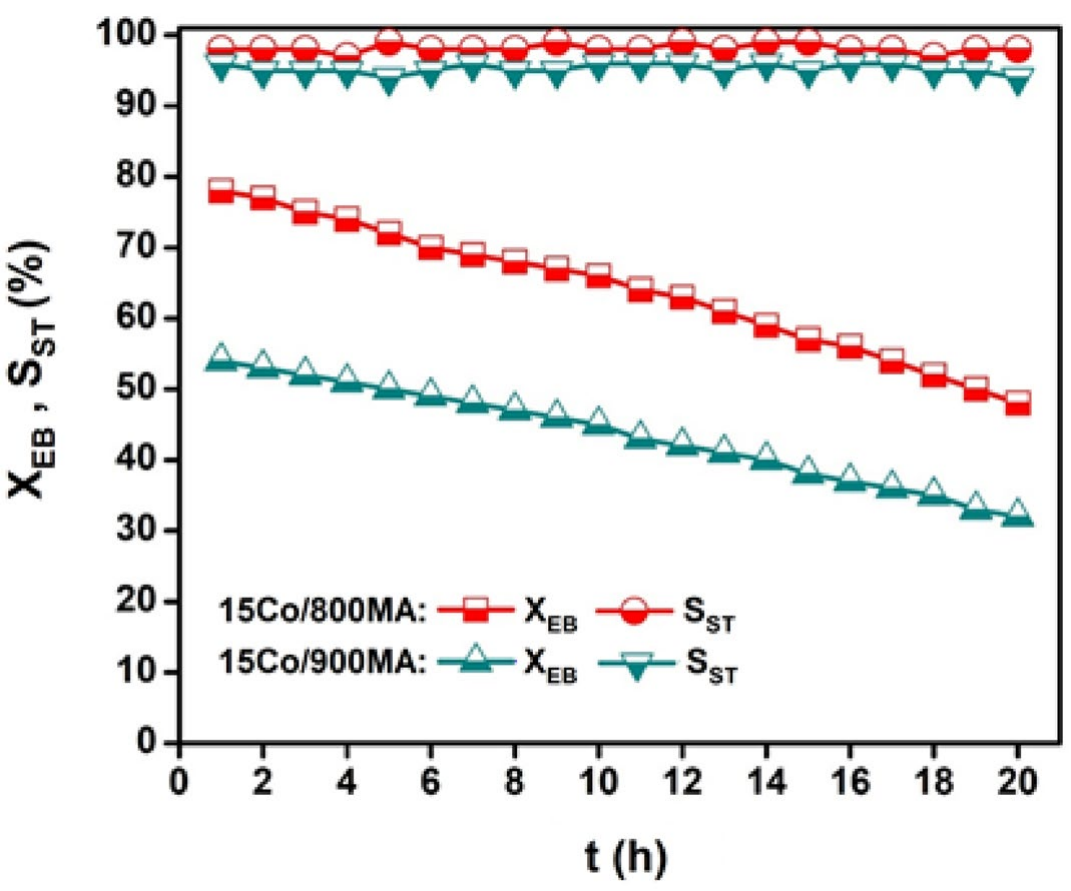
TOS on regenerated cobalt catalysts under CO_2_ flow (reaction conditions: T = 600 °C, catalyst = 0.7 g, CO_2_ flow rate = 30 mL/min, EB flow rate = 1.5 mL/h).

The regeneration of the used catalyst was performed on 15Co/800MA and 15Co/900MA samples, the corresponding results are as shown in. Typically, before the reusability studies, the specimens were activated in the airflow (30 mL/min) for 1 h. There is no observable change in the activity is observed and so we can assume that the 15Co_3_O_4_/MgAl_2_O_4_ catalyst possesses excellent thermal stability. The enhanced catalytic activity in the active presence of CO_2_ is due to the potential elimination of H_2_ formed in the dehydrogenation via reverse water–gas shift (RWGS) reaction. The amount of CO liberated during ODH of EB was accurately measured and graphically represented in Fig. S9 (supplementary information). High CO yield (≥ 9%) is obtained on 15Co/800MA catalyst compared to H_2_ evolution, which specifies the great participation of H_2_ in the RWGS reaction. While low yield of CO (6.4%) on 15Co/900MA catalyst indicates the low RWGS activity which is responsible for the mild X_EB_ (54%) as illustrated in Fig. 8. Accordingly, more fractions of carbonaceous material deposition tend to oxidize for the generation of excess CO_2_ molecules on the surface of 15Co/800MA catalyst compared to that of 15Co/900MA catalyst.

### Assessment of CO_2_ assisted ODH of EB activity of 15Co/800MA with the reported catalysts

A comparison of CO_2_ assisted oxidative dehydrogenation of EB activity over Co_3_O_4_ supported on the MgAl_2_O_4_ spinel system with the previously reported catalysts is summarized in Table 4. Guo et al*.* reported the use of a multi-walled CNT supported Co and Ni catalyst (CNT-Co-10) for the ODH of EB using CO_2_ as oxidant with a maximum X_EB_ of 83% and S_ST_ of 88% at 550 °C (Table 4, entry 1). Similarly, Braga et al*.* synthesized a CoFeSi sample by polymeric precursor method which represents a X_EB_ value of 45.5% with an affordable S_ST_ of 98% at 550 °C (Table 4, entry 2). Moronta et al*.* prepared the CoMo bimetallic catalysts supported on montmorillonite Al-pillared clay and tested for the EB dehydrogenation activity. The reduced catalysts have shown more activity than unreduced samples with a X_EB_ of 20% and S_ST_ of 91% at 400 °C (Table 4, entry 3). Burri et al*.* explored the potential application of TiO_2_-ZrO_2_ binary oxide for efficient usage of CO_2_ as a mild oxidant in the ODH of EB. The alkali promoted mixed oxide (K_2_O/ TiO_2_–ZrO_2_) displayed a high selectivity of ST (99%) with a X_EB_ of 65.5% at 650 °C (Table 4, entry 4). Correspondingly, Burri et al*.* have also investigated the application of MnO_2_-ZrO_2_ mixed metal oxide for the ODH of EB in the active presence of CO_2_. The binary metal oxide catalytic system providing a more specific surface area typically shown 73% X_EB_ and 98% S_ST_ at 650 °C (Table 4, entry 5). Madhavi et al*.* have examined the Co-Mo nitride catalysts (Co_3_Mo_3_N) for the ODH of EB using CO_2_ as mild oxidant which exhibits X_EB_ of 62.5% and S_ST_ of 94.3% (Table 4, entry 6). Pratap et al*.* reported MgAl_2_O_4_ supported CeO_2_ catalysts with high specific surface area and improved redox properties by co-precipitation method. The synthesized ceria catalysts exhibited a X_EB_ of 82% and S_ST_ of 98% at 600 °C (Table 4, entry 7). Likewise, Zhange et al*.* also investigated the high-surface-area CeO_2_ for the CO_2_ aided ODH of EB and achieved a noticeable improvement in the catalytic activity (Table 4, entry 8). Madduluri et al*.* reported the application of La_2_O_3_ incorporated Co_3_O_4_/MgO catalysts for the highly selective ODH of EB in CO_2_ with a X_EB_ of 62% (Table 4, entry 9). It is clearly evident the 15Co/800MA achieved a remarkable enhancement in the EB dehydrogenation activity with a maximum X_EB_ of 82% and S_ST_ of 98% (Table 4, entry 10), which is much superior and better than the other catalysts stated.

**Table 4:** Comparison of CO_2_ assisted EB dehydrogenation activity of 15Co/800MA with the reported catalysts.

S. No.	Catalyst	T (°C)^a^	X_EB_ (%)	S_ST_ (%)	References
1	CNT-Co-10	550	83	88.1	^19^
2	CoFeSi	550	47.5	98.0	^20^
3	CoMo/Al-ST	400	20	91	^15^
4	K_2_O/TiO_2_–MnO_2_	650	65.5	99	^10^
5	MnO_2_–ZrO_2_	650	73	98	^11^
6	Co_3_Mo_3_N	600	62.5	94.3	^22^
7	CeO_2_/MgAl_2_O_4_	450	82	98	^26^
8	CeO_2_-HSA	500	78	90	^12^
9	Co/La/MgO	600	62	99	^18^
10	15Co/800MA	600	82	98	Present study

^a^Reaction temperature for the ODH of ethylbenzene in the presence of CO_2_.

## Conclusions

In summary, 15Co_3_O_4_/MgAl_2_O_4_ spinel is found to be an efficient catalyst system for the ODH of EB in the presence of CO_2_ as a soft oxidant. Besides, the catalyst possesses remarkable stability with a prolonged activity during 20 h TOS study. A gradual decrease in the conversion from 82 to 59% is anticipated for the mild coke formation. However, the selectivity of the styrene monomer (98%) has stayed almost the same throughout the reaction. The significant improvement can be ascribed to the calcination of MgAl_2_O_4_ spinel at 800 °C, which facilitates a chemical uniformity for the distribution of Co_3_O_4_ nanoparticles on the active surface of the catalyst. To be specific, a greater number of uniform Co_3_O_4_ oxide particles on the 800MA spinel results in the high specific surface area and optimum crystallite size responsible for the enhancement in the catalytic activity. Furthermore, the coke formation was suppressed effectively by the oxidation of CO_2_ as a soft oxidant. It is supposed to be due to the promotion of RWGSR in the CO_2_ environment, which performed a crucial role to achieve the maximum catalytic activity.

## Supplementary information

Supplementary Information

## Data Availability

The datasets generated and analyzed during the current study are included in this article and also it is available from the corresponding author on reasonable request.
