# Supplementary material for: CO2 utilization as a soft oxidant for the synthesis of styrene from ethylbenzene over Co3O4 supported on magnesium aluminate spinel: role of spinel activation temperature
Source: Sci Rep. 2020 Dec 17;10:22170. doi: 10.1038/s41598-020-79188-z (PMC7747735; doi:10.1038/s41598-020-79188-z)
Supplement: Supplementary file 1 — Supplementary Information [file 41598_2020_79188_MOESM1_ESM.docx]

**Supplementary information**

**CO_2_ utilization as a soft oxidant for the synthesis of styrene from ethylbenzene over Co_3_O_4_ supported on magnesium aluminate spinel: role of spinel activation temperature**

Venkata Rao Madduluri,^1,⸸,*^ Ravi Kumar Marella,^1,2,⸸^ Marlia M. Hanafiah,^3,4^ Sivarama Krishna Lakkaboyana,^5,*^ G. Suresh babu^6^

^1^Catalysis and Fine Chemicals Laboratory, Indian Institute of Chemical Technology, Hyderabad, 500007, India.

*^2^*Department of Chemistry (H & S), PACE Institute of Technology & Sciences, Ongole 523001, Andhra Pradesh, India.

^3^Department of Earth Sciences and Environment, Faculty of Science and Technology, Universiti Kebangsaan Malaysia, 43600 UKM Bangi, Selangor, Malaysia.

^4^Centre for Tropical Climate Change System, Institute of Climate Change, Universiti Kebangsaan Malaysia, 43600 UKM Bangi, Selangor, Malaysia.

^5^School of Ocean Engineering, Universiti Malaysia Terengganu, 21030, Kuala Nerus, Terengganu Darul Iman, Malaysia.

^6^Academy of Scientific and Innovative Research (AcSIR), Sector 19, Kamala Nehru Nagar, Ghaziabad 20100, Uttar Pradesh, India

*Corresponding author. E-mail: [*mvrjntu@gmail.com*](mailto:mvrjntu@gmail.com%20) (V.R.M), *[svurams@gmail.com](mailto:svurams@gmail.com)* *(*S.K.L)

^⸸^these authors contributed equally to this work

**Catalytic tests**

The catalytic ODH of EB was conducted in a fixed bed downflow quartz reactor (14 mm I.D. and 450 mm long) at atmospheric pressure in the temperature range of 450-650 °C under CO_2_ and N_2_ flow. About 0.7 g of the catalyst diluted with quartz beads was loaded in the middle of the reactor. The reactor was placed electrically connected vertically opened hot furnace and temperature is controlled by a programmer connected with a K-type thermocouple. The catalyst bed is heated at 650 °C in N_2_ flow (30 mL/min) for 1 h prior to the reaction. After the pre-heating, the temperature was switched to the required reaction atmosphere, and the experiment was carried out by passing EB (1.5mL/h) with either N_2_ or CO_2_ gas (30 mL/min). The reaction mixture was collected in an ice-cold glass trap every 1 h and analyzed by the Agilent 7890A GC equipped with FID using EB-5 capillary column. The gaseous products (CO, CH_4_, CO_2_, and H_2_) are detected through online gas analysis by TCD detector using the Porapak-Q column. The fractional conversion of ethylbenzene (X_EB_), yield of styrene (Y_ST_), and its selectivity (S_ST_) can be expressed using the following equations.

| $X_{\mathrm{EB}} \left( \% \right)=\frac{\left( \mathrm{EB} \right)_{in}-\left( \mathrm{EB} \right)_{out}}{\left( \mathrm{EB} \right)_{in}}\times100 \left( 1 \right)$ |
| --- |
| $Y_{\mathrm{ST}} (\%)=\frac{\left( \mathrm{ST} \right)_{out}}{\left( \mathrm{EB} \right)_{in}}\times100$ (2) |
| $S_{\mathrm{ST}} (\%)=\frac{\left( Y_{\mathrm{ST}} \right)}{\left( X_{\mathrm{EB}} \right)}\times100$ (3) |



**Fig. S1** XRD patterns of (a) 10Co/800MA and (b) 20Co/800MA catalysts


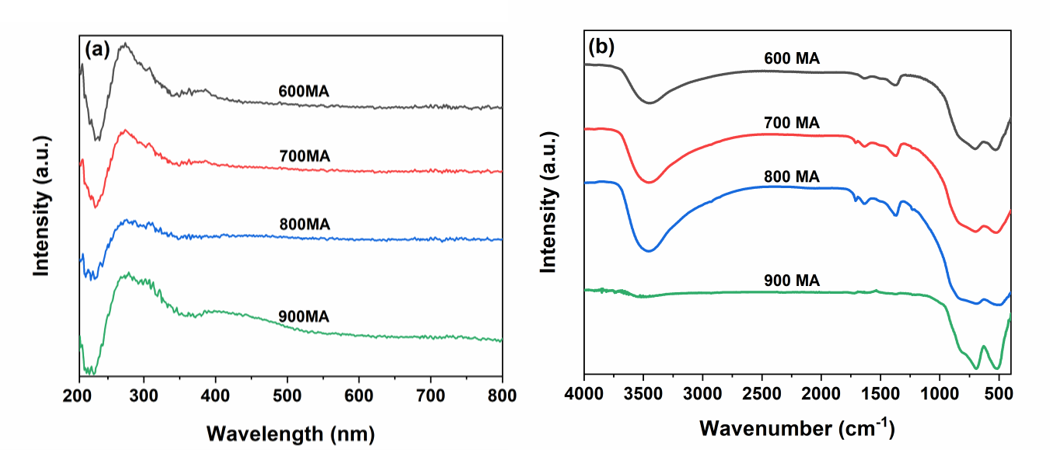


**Fig. S2** (a) UV-Vis DRS and (b) FTIR spectra of MgAl_2_O_4_ at various activation temperatures


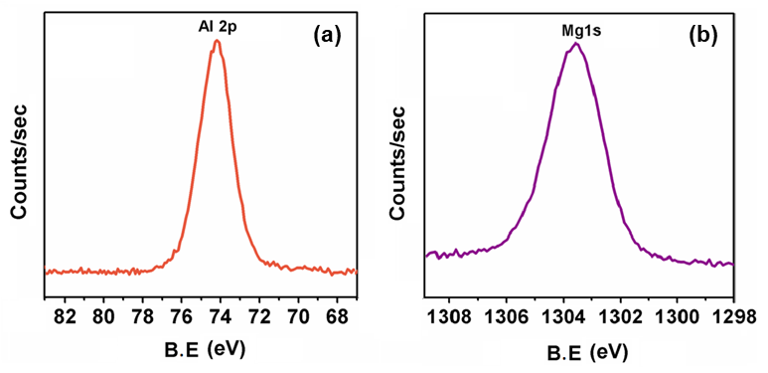
**Fig. S3** (a) Al 2p and (b) Mg 1s XPS of 15Co/800MA catalyst

**
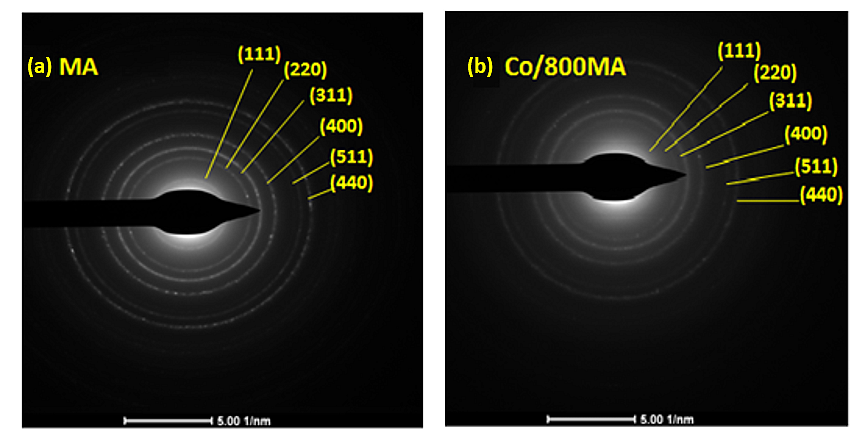
**

**Fig. S4** SAED images of (a) 800MA spinel and (b) 15Co/800MA catalyst


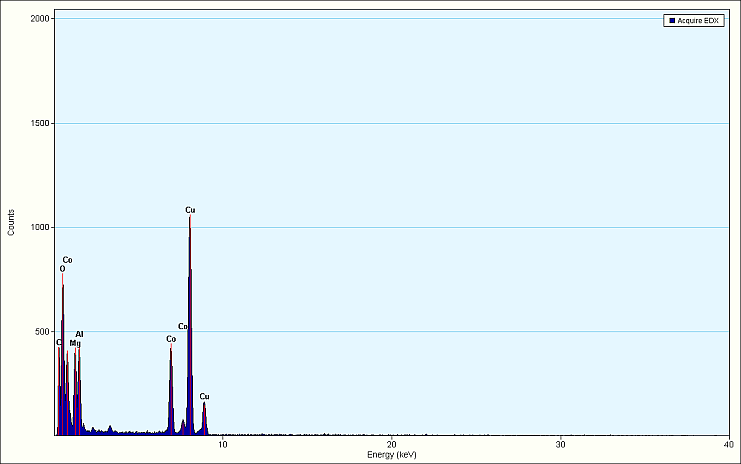
**Fig. S5** EDS spectra of fresh 15Co/800MA catalyst


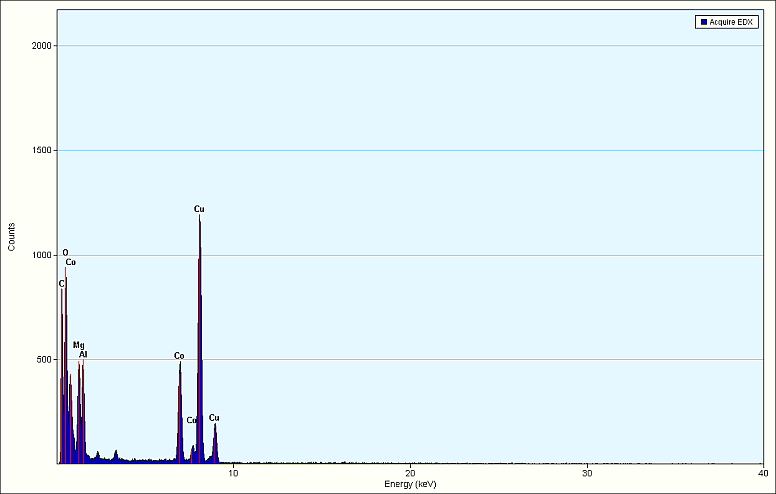


**Fig. S6** EDS spectra of spent 15Co/800MA catalyst


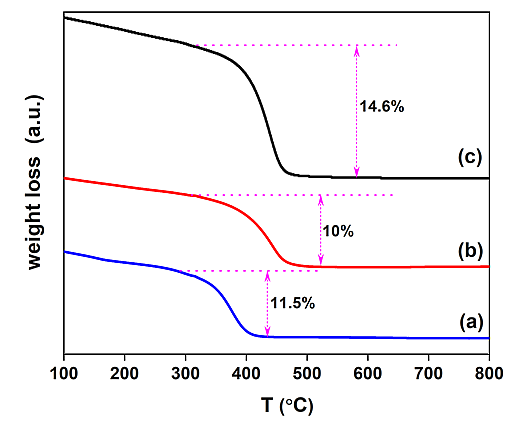
**Fig. S7** TG analysis of spent catalysts (a) 15Co/700MA, (b) 15Co/800MA and (c) 15Co/900MA


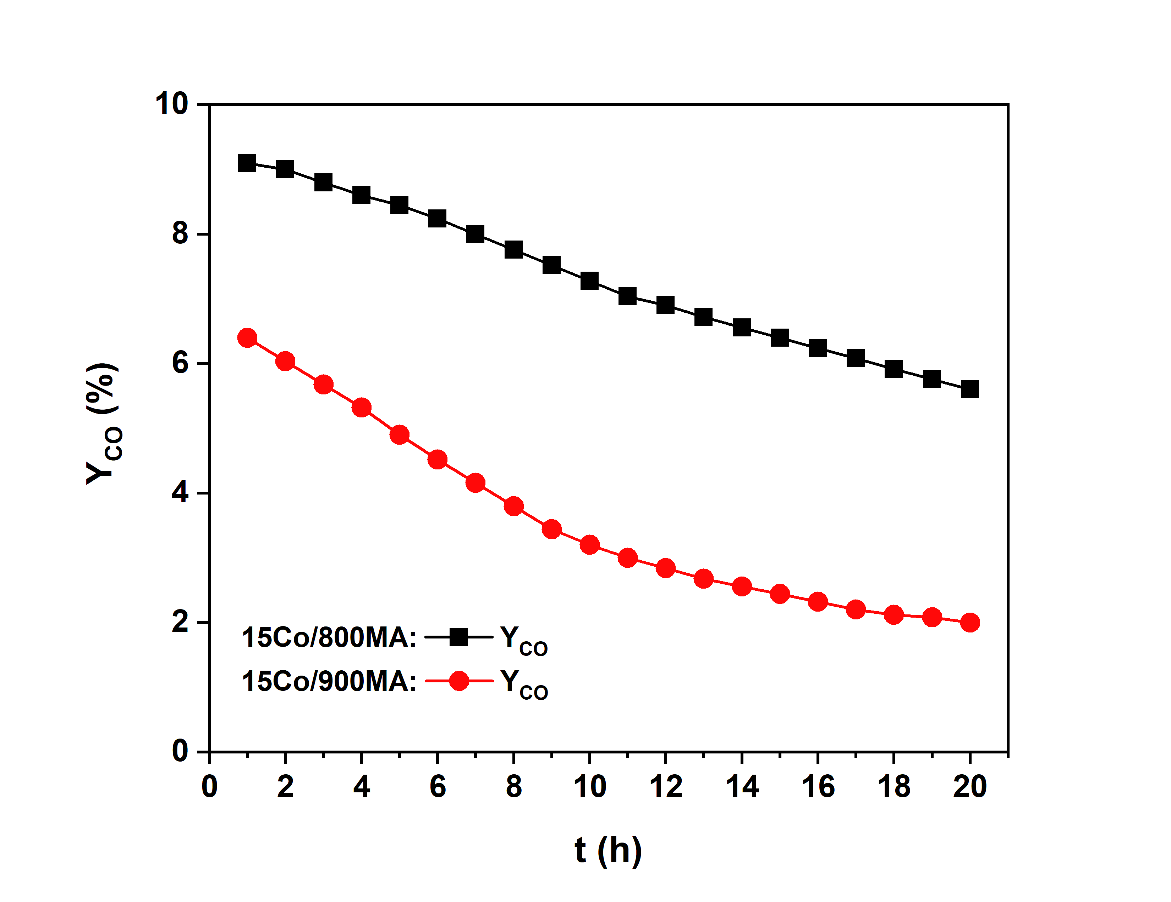
**Fig. S8** Yield of CO over 15Co/800MA and 15Co/900MA catalysts under CO_2_ flow (Reaction conditions: T = 600 °C, catalyst = 0.7g, CO_2_ flow rate = 30 ml/min, EB flow rate = 1.5ml/h)
